# Stereotactic Localization: From Single-Slice to Multi-Slice Registration Including a Novel Solution for Parallel Bipanels

**DOI:** 10.7759/cureus.23279

**Published:** 2022-03-17

**Authors:** Mark Sedrak, Andres E Bruna, Armando L Alaminos-Bouza

**Affiliations:** 1 Neurosurgery, Kaiser Permanente, Redwood City, USA; 2 Medical Physics, Fisica Médica SRL, Córdoba, ARG; 3 Medical Physics, MEVIS Informática Médica Ltda., São Paulo, BRA

**Keywords:** frame-based stereotactic surgery, localization, stereotactic and functional neurosurgery, functional neurosurgery, stereotactic frame

## Abstract

Frame-based stereotactic localization generally assumes that all required fiducials are present in a single-slice image which can then be used to form targeting coordinates. Previously, we have published the use of novel localizers and mathematics that can improve stereotactic localization. As stereotactic procedures include numerous imaging slices, we sought to investigate, develop, and test techniques that utilize multiple slices for stereotactic localization and provide a solution for a parallel bipanel N-localizer.

Several multi-slice equations were tested. Specifically, multi-slice stereotactic matrices (ms-SM) and multi-slice normal to parallel planes (ms-nPP) were of particular interest. Bipanel (2N) and tripanel (3N) localizer images were explored to test approaches for stereotactic localization. In addition, combination approaches using single-slice stereotactic matrices (ss-SM) and multi-slice methods were tested.

Modification of ss-SM to form ms-SM was feasible. Likewise, a method to determine ms-nPP was developed. For the special case of the parallel bipanel N-localizer, single-slice and multi-slice methods fail, but a novel non-linear solution is a robust solution for ms-nPP.

Several methods for single-slice and multi-slice stereotactic localization are described and can be adapted for nearly any stereotactic system. It is feasible to determine ms-SM and ms-nPP. In particular, these methods provide an overdetermined means to calculate the vertical z, which is determined for a tripanel system using single-slice methods. In addition, the multi-slice methods can be used for extrapolation outside of the localizer space. Importantly, a novel non-linear solution can be used for parallel bipanel N-localizer systems, where other methods fail. Finally, multi-slice stereotactic localization assumes strict patient and imaging system stability, which should be carefully assessed for each case.

## Introduction

Frame-based stereotactic localization is generally constructed for single-slice image localization [1-8]. As such, each image contains all necessary fiducials required to compute single-slice stereotactic matrices (ss-SM), which allow for conversion between two-dimensional (2D) screen coordinates and three-dimensional (3D) stereotactic frame-based coordinates for that image slice.

Previously, we have proposed several optimizations to the ss-SM that can reduce error [2]. Specifically, the use of overdetermined systems of equations using all fiducial data improves the solution to minimize errors. However, single-slice localization ignores data in adjacent slices, which, in a stable system, can be added to further improve solutions. Using a three-localizer system, the use of overdetermined ss-SM reduces errors only for the x- and y- axes, not for the z-axis. Therefore, it could be desirable to add data regarding the z-axis to minimize errors. Also, ss-SM cannot be used for extrapolation to adjacent slices. Therefore, we sought to develop multi-slice solutions to provide additional solutions for which ss-SM are limited. 

## Technical report

Stereotactic imaging

**Figure 1:**
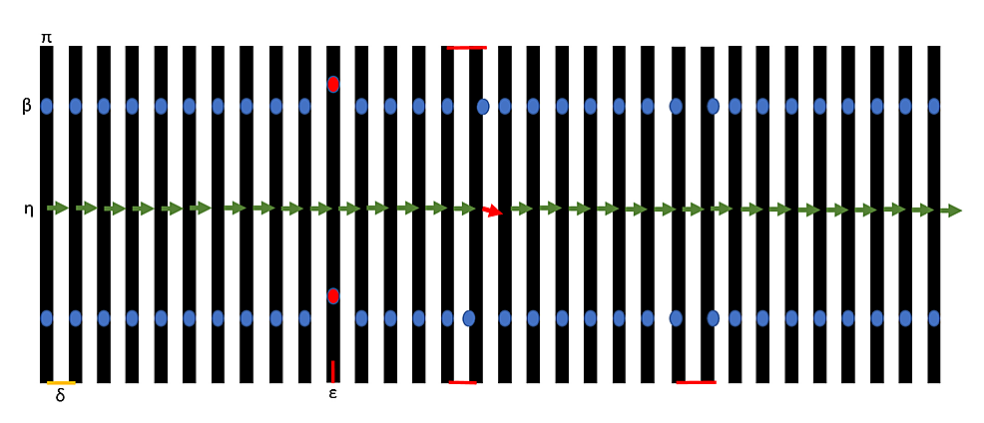
A stereotactic volume of image slices Stereotactic imaging includes the use of computed tomography (CT) and magnetic resonance imaging (MRI) for image acquisition. Each one of these images can be considered a mathematical plane $\pi$ (black). These images, in frame-based stereotaxis, include fiducials $\beta$, where the vertical rods are represented (blue). Using these fiducials, such as for N-localizer or Sturm-Pastyr localizers, the normal vector to the plane $\eta$ can be computed (green). Each image in the stack is separated by a distance from plane-to-plane $\delta$ (yellow), which may be assumed to be a constant. Finally, errors $\varepsilon$ (red) can be seen when these assumptions are not correct, causing displacement of $\beta$, $\delta$ and/or $\eta$. This illustrative image exaggerates and may not fully represent potential problems with image slices.

Stereotactic imaging includes the use of computed tomography (CT) and magnetic resonance imaging (MRI) for image acquisition to acquire a series of parallel images. Each image slice can be considered a mathematical plane $\pi$ (Figure 1). These images, in frame-based stereotaxis, include fiducials $\beta$, where the vertical rods are represented in Figure 1. Using these fiducials, such as for N- or Sturm-Pastyr localizers, the normal vector to each plane $\eta$ can be computed when three or more 3D points are present in a slice. When considering ss-SM, each image contains all relevant data. However, when considering multi-slice calculations, there are assumptions about stability from slice-to-slice. Adjacent images in the stack are separated by a distance from slice-to-slice $\delta$. Unexpected errors $\varepsilon$ may occur in adjacent slices and can be seen in Figure 1 as $\beta$, $\delta$ and $\eta$. These errors need to be considered with multi-slice solutions.

The following sections summarize important techniques for stereotactic localization as it relates to single- or multi-slice techniques. As previously published, linear methods for using an overdetermined system of equations are emphasized in the mathematics presented [2], but a non-linear solution also emerges. Stereotactic matrices are the basis for stereotactic localization, but we also utilize a plane equation which we extend from a single- to multi-slice application. In particular, the multi-slice plane calculation leads us to a new novel non-linear solution that is needed for a parallel bipanel system. For reference, in the subsequent sections, we refer to some of the matrices loosely as forward or reverse solutions. Also, some of these matrix solutions can be parsed into x-axis, y-axis, and/or z-axis dimensions (e.g., X, Y, Z, XY, XYZ). 

Single-slice stereotactic matrices (ss-SM)

As previously published, the single-slice stereotactic matrix (ss-SM) affords a critical role in frame-based stereotactic neurosurgery [1-2]. The ss-SM can be computed using equation 1 or equation 2, which transform 2D coordinates to 3D, or vice versa, for a given slice. The matrices, rearranged as a 3x3 matrix (equation 3), incorporate rotation, scaling, and translation for an affine transformation, where $^{-1}$ refers to the inverse matrix. These ss-SM can be computed using a determined dataset, the minimum number of variables needed to solve the unknowns ($m, t$), or overdetermined, greater than the minimum number of variables needed to solve the unknowns. Again, using ss-SM, one can convert 2D coordinates ($u, v$) to or from 3D coordinates ($x, y, z$) (equation 4, 5). It should be noted that ss-SM is computed for each slice independently and is not influenced by adjacent slices, as seen in Figure 1.

$$\begin{bmatrix} u_{1} & v_{1} & 1 & 0 & 0 & 0 & 0 & 0 & 0 \\ 0 & 0 & 0 & u_{1} & v_{1} & 1 & 0 & 0 & 0\\ 0 & 0 & 0 & 0 & 0 & 0 & u_{1} & v_{1} & 1 \\ \vdots & & & & & & & & \vdots\\ u_{n} & v_{n} & 1 & 0 & 0 & 0 & 0 & 0 & 0 \\ 0 & 0 & 0 & u_{n} & v_{n} & 1 & 0 & 0 & 0\\ 0 & 0 & 0 & 0 & 0 & 0 & u_{n} & v_{n} & 1 \end{bmatrix} \cdot \begin{bmatrix} m_{11}\\ m_{21}\\ m_{31} \\ m_{12}\\ m_{22}\\ m_{32} \\m_{13}\\ m_{23}\\ m_{33} \end{bmatrix} = \begin{bmatrix} x_{1}\\ y_{1}\\ z_{1} \\ \vdots\\ x_{n}\\ y_{n} \\ z_{n} \end{bmatrix}\tag{1}$$

$$\begin{bmatrix} x_{1} & y_{1} & z_{1} & 0 & 0 & 0 & 0 & 0 & 0 \\ 0 & 0 & 0 & x_{1} & y_{1} & z_{1}& 0 & 0 & 0\\ 0 & 0 & 0 & 0 & 0 & 0 & x_{1} & y_{1} & z_{1}\\ \vdots & & & & & & & & \vdots\\ x_{n} & y_{n} & z_{n}& 0 & 0 & 0 & 0 & 0 & 0 \\ 0 & 0 & 0 & x_{n} & y_{n} & z_{n} & 0 & 0 & 0\\ 0 & 0 & 0 & 0 & 0 & 0 & x_{n} & y_{n} & z_{n} \end{bmatrix} \cdot \begin{bmatrix} t_{11}\\ t_{21}\\ t_{31} \\ t_{12}\\ t_{22}\\ t_{32} \\ t_{13}\\ t_{23}\\ t_{33} \end{bmatrix} = \begin{bmatrix} u_{1}\\ v_{1}\\ 1 \\ \vdots\\ u_{n}\\ v_{n} \\ 1 \end{bmatrix}\tag{2}$$

$$\begin{bmatrix} m_{11} & m_{12} & m_{13} \\ m_{21} & m_{22} & m_{23}\\ m_{31} & m_{32} & m_{33} \end{bmatrix} = \begin{bmatrix} t_{11} & t_{12} & t_{13} \\ t_{21} & t_{22} & t_{23}\\ t_{31} & t_{32} & t_{33} \end{bmatrix}^{-1} \tag{3}$$

$$\begin{bmatrix} u_{i} & v_{i} & 1 \end{bmatrix} \cdot \begin{bmatrix} m_{11} & m_{12} & m_{13} \\ m_{21} & m_{22} & m_{23}\\ m_{31} & m_{32} & m_{33} \end{bmatrix} = \begin{bmatrix} x_{i} & y_{i} & z_{i} \end{bmatrix}\tag{4}$$

$$\begin{bmatrix} x_{i} & y_{i} & z_{i} \end{bmatrix} \cdot \begin{bmatrix} t_{11} & t_{12} & t_{13} \\ t_{21} & t_{22} & t_{23}\\ t_{31} & t_{32} & t_{33} \end{bmatrix} = \begin{bmatrix} u_{i} & v_{i} & 1 \end{bmatrix}\tag{5}$$

Single-slice normal to a plane (ss-nP)

The normal vector $\vec n$ to a plane can be computed for each individual slice when three or more 3D points are present in a given image slice. This normal ( $\vec n$) is a vector that is perpendicular to the plane and defines its slope. Given three 3D points, $P, Q, R$, one can compute the $\vec n$ by using the cross-product ($\times$) of two vectors ($P -Q$ and $R-Q$) formed by the three points (equation 6). This three-point solution is a determined solution, containing the minimum requirement for determining $\vec n$. To generalize this into an overdetermined system of linear equations for single-slice normal to a plane (ss-nP), we first analyze the equation of a 3D plane (equation 7). The normal to the plane ($\vec n$) is represented by coefficients $a, b, c$, whereas $x, y, z$ are variable 3D coordinates and $d$ serves as a translation coefficient. To set up the linear equation, we first take $cz$ and move them to the right side of the equation (equation 8), although this could be accomplished similarly with either $ax$ or $by$, which may be useful for non-axial slices. A system of linear equations can now be set up, solving for $a, b, d$ while keeping $c=1$ (equation 9). It may be useful to keep $a,b,c$ values in unit vector form $\hat n$ by dividing by the magnitude of $\vec n$ (equation 10). The rearrangement of equation 7 allows a solution for $d$, which can be set up in matrix form to minimize errors (equation 11). Also, $z$ can be solved using $a, b, c, d$ along with $x, y$ anywhere in the plane (equation 12). It should be noted that ss-nP is computed for each slice independently and is not influenced by adjacent slices, as seen in Figure 1.

$$\vec n = (P-Q) \times (R-Q) \tag{6}$$

$$ax + by + cz + d = 0 \tag{7}$$

$$ax + by + d = -cz \tag{8}$$

$$\begin{bmatrix} x_{1} & y_{1} & 1 \\ \vdots && \vdots\\ x_{n} & y_{n} & 1 \end{bmatrix} \cdot \begin{bmatrix} a\\ b\\ d \end{bmatrix} = \begin{bmatrix} -cz_{1} \\ \vdots\\ -cz_{n} \end{bmatrix}\tag{9}$$

$$\hat n = \frac{ \vec n} { \vert \vec n \vert} \tag{10}$$

$$\begin{bmatrix} 1 \\ \vdots \\ 1 \end{bmatrix} \cdot \begin{bmatrix} d \end{bmatrix} = \begin{bmatrix} -(ax_{1} + by_{1} + cz_{1}) \\ \vdots \\ -(ax_{i} + by_{i} + cz_{i}) \end{bmatrix}\tag{11}$$

$$z = \frac { -ax -by-d} {c}\tag{12}$$

Multi-slice normal to parallel planes (ms-nPP)

A solution for a normal vector that is a multi-slice normal to parallel planes (ms-nPP) would inherently be an overdetermined solution utilizing information over the imaging volume. Assuming that multi-slice registration includes planes that are all parallel, we start with the equation of a 3D plane (equation 7). However, given that we have parallel planes, we now need to create a variable within the translation $d$ by making $se + f$ (equation 13). This slice position $s$ for equal sliced image sequences can best be represented in using Digital Imaging and Communications in Medicine (DICOM) tags, such as the image position patient (0020,0032), image location attribute (0020, 1041), image spacing attribute (0018,0088), or a $z$ or average $z$ of the diagonal rods, for example. Caution should be used when using DICOM metadata as there can be device variability. Therefore, $d$ can now be computed for each image slice in the whole image volume using variables $s, e, f$. In matrix form, the equation is setup using the parallel planes modification of the 3D plane equation (equation 14). To facilitate the solution, which needs to be solved for $a, b, e, f$, we can set $c = 1$ or to any non-zero value. Therefore, the normal to the planes ($\vec n$) solution includes the variables $a, b, c$, where $c = 1$. Using $\vec n$, one can now solve for $d$ in any slice in the volume using one or more 3D ($x, y, z$) points. Then, having $a, b, c, d$, for any $x, y$ point, such as for the vertical rods, the vertical $z$ can then be computed using equation 11. It should be noted that ms-nPP is computed using many slices and can be influenced by adjacent slices, as seen in Figure 1.

$$ax + by + es + f = -cz \tag{13}$$

$$\begin{bmatrix} x_{1} & y_{1} & s_{1} & 1 \\ \vdots &&& \vdots\\ x_{n} & y_{n} & s_{n} & 1 \end{bmatrix} \cdot \begin{bmatrix} a\\ b\\ e \\ f \end{bmatrix} = \begin{bmatrix} -cz_{1} \\ \vdots\\ -cz_{n} \end{bmatrix}\tag{14}$$

Multi-slice stereotactic matrices (ms-SM)

As observed in multi-slice parallel planes, a multi-slice stereotactic matrix (ms-SM) requires the variability of a slice. Given that 2D screen coordinates are generally known with a given slice, the variable $s$ is used along with $u, v$. Taking equation 1, we now introduce $s$ and expand the column vector matrix $m$ to twelve variables rather than nine (equation 15). As in equation 2, the inverse matrix $t$ can be accomplished by changing the variables around (equation 16). Finally, matrix $m$ and $t$ are relatable, where $^{-1}$ refers to the inverse matrix (equation 17). Using these multi-slice stereotactic matrices, one can then convert $u, v, s$ coordinates to $x, y, z$ coordinates (equation 18) or vice versa (equation 19). It should be noted that ms-SM is computed using many slices and can be influenced by adjacent slices, as seen in Figure 1.

$$\begin{bmatrix} u_{1} & v_{1} & s_{1} & 1 & 0 & 0 & 0 & 0 & 0 & 0 & 0 & 0\\ 0 & 0 & 0 & 0 & u_{1} & v_{1} & s_{1} & 1 & 0 & 0 & 0 & 0 \\ 0 & 0 & 0 & 0 & 0 & 0 & 0 & 0 & u_{1} & v_{1} & s_{1} & 1 \\ \vdots & & & & & & & & & & & \vdots\\ u_{n} & v_{n} & s_{n} & 1 & 0 & 0 & 0 & 0 & 0 & 0 & 0 & 0 \\ 0 & 0 & 0 & 0 & u_{n} & v_{n} & s_{n} & 1 & 0 & 0 & 0 & 0 \\ 0 & 0 & 0 & 0 & 0 & 0 & 0 & 0 & u_{n} & v_{n} & s_{n} & 1 \end{bmatrix} \cdot \begin{bmatrix} m_{1}\\ m_{2}\\ m_{3} \\ m_{4}\\ m_{5}\\ m_{6} \\m_{7}\\ m_{8} \\ m_{9}\\ m_{10} \\m_{11}\\ m_{12} \end{bmatrix} = \begin{bmatrix} x_{1}\\ y_{1}\\ z_{1} \\ \vdots\\ x_{n}\\ y_{n} \\ z_{n} \end{bmatrix}\tag{15}$$

$$\begin{bmatrix} x_{1} & y_{1} & z_{1} & 1 & 0 & 0 & 0 & 0 & 0 & 0 & 0 & 0\\ 0 & 0 & 0 & 0 & x_{1} & y_{1} & z_{1} & 1 & 0 & 0 & 0 & 0 \\ 0 & 0 & 0 & 0 & 0 & 0 & 0 & 0 & x_{1} & y_{1} & z_{1} & 1 \\ \vdots & & & & & & & & & & & \vdots\\ x_{n} & y_{n} & z_{n} & 1 & 0 & 0 & 0 & 0 & 0 & 0 & 0 & 0 \\ 0 & 0 & 0 & 0 & x_{n} & y_{n} & z_{n} & 1 & 0 & 0 & 0 & 0 \\ 0 & 0 & 0 & 0 & 0 & 0 & 0 & 0 & x_{n} & y_{n} & z_{n} & 1 \end{bmatrix} \cdot \begin{bmatrix} t_{1}\\ t_{2}\\ t_{3} \\ t_{4}\\ t_{5}\\ t_{6} \\t_{7}\\ t_{8} \\ t_{9}\\ t_{10} \\t_{11}\\ t_{12} \end{bmatrix} = \begin{bmatrix} u_{1}\\ v_{1}\\ s_{1} \\ \vdots\\ u_{n}\\ v_{n} \\ s_{n} \end{bmatrix}\tag{16}$$

$$\begin{bmatrix} m_{1} & m_{5} & m_{9} &0 \\ m_{2} & m_{6} & m_{10} &0\\ m_{3}& m_{7} & m_{11} &0 \\ m_{4} & m_{8}& m_{12} &1 \end{bmatrix} = \begin{bmatrix} t_{1} & t_{5} & t_{9} &0 \\ t_{2} & t_{6} & t_{10} &0\\ t_{3}& t_{7} & t_{11} &0 \\ t_{4} & t_{8}& t_{12} &1 \end{bmatrix}^{-1} \tag{17}$$

$$\begin{bmatrix} u_{i} & v_{i} & s_{i} & 1 \end{bmatrix} \cdot \begin{bmatrix} m_{1} & m_{5} & m_{9} \\ m_{2} & m_{6} & m_{10} \\ m_{3}& m_{7} & m_{11} \\ m_{4} & m_{8}& m_{12} \end{bmatrix} = \begin{bmatrix} x_{i} & y_{i} & z_{i} \end{bmatrix}\tag{18}$$

$$\begin{bmatrix} x_{i} & y_{i} & z_{i} & 1 \end{bmatrix} \cdot \begin{bmatrix} t_{1} & t_{5} & t_{9} \\ t_{2} & t_{6} & t_{10} \\ t_{3}& t_{7} & t_{11} \\ t_{4} & t_{8}& t_{12} \end{bmatrix} = \begin{bmatrix} u_{i} & v_{i} & s_{i} \end{bmatrix}\tag{19}$$

Non-linear solution for multi-slice normal to parallel planes 

Generally speaking, a linear solution is preferred when the mathematical model makes that possible. However, there are many occasions where linear solutions cannot be used directly. Further, many of the aforementioned solutions will not function in the ill-conditioned parallel bipanel. To resolve this issue, let us start by rearranging values $es$ (equation 20, 21). We will explore this further below. 

$$ax + by + f = -cz -es\tag{20}$$

$$\begin{bmatrix} x_{1} & y_{1} & 1 \\ \vdots & & \vdots\\ x_{n} & y_{n} & 1 \end{bmatrix} \cdot \begin{bmatrix} a\\ b \\ f \end{bmatrix} = \begin{bmatrix} (-cz_{1} - es_{1}) \\ \vdots\\ (-cz_{n} - es_{n}) \end{bmatrix}\tag{21}$$

**Figure 2:**
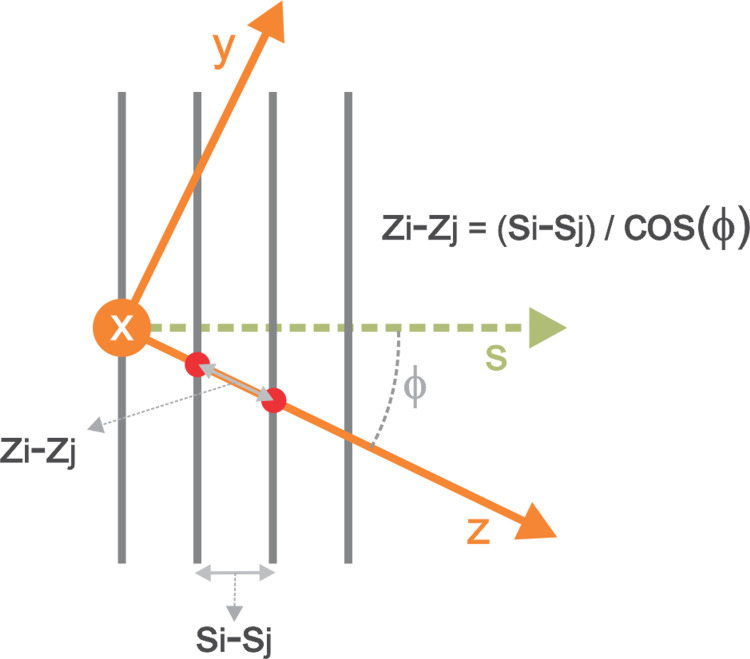
Graphical interpretation of $z$ and $s$ for multi-slice normal to parallel planes computation relative to x-, y-, and z-axes The value of $z_{i}-z_{j}$ are $z$ values along each image slice (black vertical lines), whereas $s_{i}-s_{j}$ are $s$ values along each image slice. When there is a rotation angle such as frame tilt $\phi$, then $z_{i}-z_{j}$ varies differently than $s_{i}- s_{j}$. When there is no rotation, then $z_{i}-z_{j}$ is equal to $s_{i}- s_{j}$.

In Figure 2, the image plane is represented by vertical blue lines, and the coordinate system to use is the stereotactic $x, y, z$, where $z$ is represented in orange color, $s$ represents the slice location coordinate on an axis normal to the images, and $\phi$ represents the angle between $z$ and $s$.

Let us now consider equation 7. Being that for multi-slice circumstances, the planes are assumed to be parallel to each other, share the same origin $x, y, z$, values of $a, b, c$, but differ in $d$. We can say that $d$ is a function of $s$ (equation 22), where $s$ can be formed from DICOM information [9]. 

$$d = d(s) \tag{22}$$

Now we consider two planes with different $s_{i}$ and $s_{j}$ values and in each of these planes the points with $x=y=0$. These may be represented as the two red dots in Figure 2. Using equation 22, we can create equation 23, and using Figure 2, we get equation 24, demonstrating that $\phi$ is related to frame tilt (rotation around the x-axis). A frame swivel angle $\theta$ (rotation around the y-axis) can also be incorporated, giving us equation 25. 

$$d(s_{i})-d(s_{j}) = c(z_{i} - z_{j}) \tag{23}$$

$$z_{i}-z_{j} = (s_{i}-s_{j})/cos(\phi) \tag{24}$$

$$z_{i}-z_{j} = (s_{i}-s_{j})/cos(\phi)cos(\theta) \tag{25}$$

Defining a constant $e$ (equation 26), we develop equation 27, which we can integrate to get equation 28 where $f$ is a constant that does not vary with $s$ nor $x, y, z$. We can now define the non-unitary normal vector $n$ (equation 29) and define $M$ (equation 30) as the magnitude/modulus (mod) of the normal vector $n$.

$$e:= \frac{c} {cos(\phi)cos(\theta)} \tag{26}$$

$$d(s_{i})-d(s_{j}) = e(s_{i}-s_{j}) \tag{27}$$

$$d(s) = es + f \tag{28}$$

$$n := (a,b,c) \tag{29}$$

$$M = \sqrt{a^2 + b^2 + c^2 }\tag{30}$$

Using trigonometry, we can rearrange equation 31 and give equation 32, 33, and arrive at equation 34, which is an important relationship if $s$ is computed correctly. 

$$c = M cos(\phi) cos(\theta) \tag{31}$$

$$cos(\phi) = \frac{c}{M cos(\theta)} \tag{32}$$

$$cos(\phi)cos(\theta) = \frac{c}{M} \tag{33}$$

$$e = M \tag{34}$$

We now take equation 20 and insert $e$ to give us equation 35. We can rearrange equation 35 to give equation 36. We now must accommodate the direction of $s$ relative to $z$ and introduce a constant $H$, which depends on the direction of $\vec s$ relative to $\vec z$ (equation 37). Finally, equation 38 is set up for non-linear solutions utilizing the value $H$ and can be facilitated by setting $c=1$. For every slice, there are two points ($x, y, z, s$) giving $n$ equations (equation 39). Finally, we set equation 40 into matrix form where the unknowns are $a, b, f$.

$$ax + by + f = -cz - s\sqrt{a^2+b^2+c^2} \tag{35}$$

$$ax + by + f + s\sqrt{a^2+b^2+c^2} = -cz \tag{36}$$

$$H := \pm 1 \tag{37}$$

$$ax + by + f + Hs\sqrt{a^2+b^2+c^2} = -cz \tag{38}$$

$$n = 2 * (number \space of \space slices) \tag{39}$$

$$\begin{bmatrix} ax_{1} + by_{1} + f + Hs_{1}\sqrt{a^2+b^2+1} \\ \vdots \\ ax_{n} + by_{n} + f + Hs_{n}\sqrt{a^2+b^2+1} \end{bmatrix} = \begin{bmatrix} -z_{1} \\ \vdots\\ - z_{n} \end{bmatrix}\tag{40}$$

**Figure 3:**
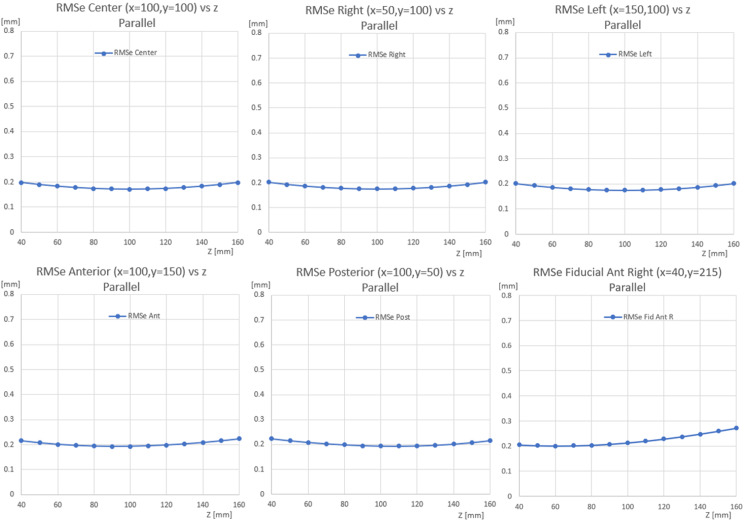
Multi-slice normal to parallel planes (ms-nPP) solution for a parallel N-localizer Here we analyzed the non-linear solution using Monte Carlo simulations and computed Root Mean Square errors (RMSe). We find that the error rates along the z-axis were low throughout the volume. Tested locations include (x,y) positions that are in the center (100,100) (top left), right (50, 100) (top middle), left (150,100) (top right), anterior (100,150) (bottom left), posterior (100,50) (bottom middle), anterior-right (40, 215) (bottom right).

While using ms-nPP equation 14, $s$ values need to represent the sequence of slices but do not need to be calibrated to the scale used in the rest of the equation (e.g., millimeter). This is because $e$ is directly solved in the linear formulation in conjunction with $s$. However, equation 38 requires that $s$ be calibrated precisely; otherwise, the results will be incorrect. Similarly, equation 21 could be linearly solved with $s$ being calibrated and with an approximation $e = \pm 1$ for a nearly orthogonal axial image set (no tilt or swivel rotation) where the normal is approximately $a, b, c \approx 0, 0, 1$. This assumption is not entirely accurate in real scenarios necessitating the non-linear solution. Further, the non-linear solution to ms-nPP has been tested in cases of significant tilt/swivel rotations and with the introduction of random noise (0-1 mm) with accurate results. Next, we computed ms-nPP via the non-linear method and ran Monte-Carlo simulations (Figure 3). Root Mean Square error (RMSe) was then computed using the solution for several positions throughout the volume. 

## Discussion

Stereotactic neurosurgery has seen continuous growth since its original development [3, 10-16]. Additionally, frame-based stereotaxis has yielded some of the most accurate operations performed in the human brain. As the field continues to evolve, improvements in the accuracy of stereotactic localization are paramount as they have inherent ramifications on surgical procedures.

**Figure 4:**
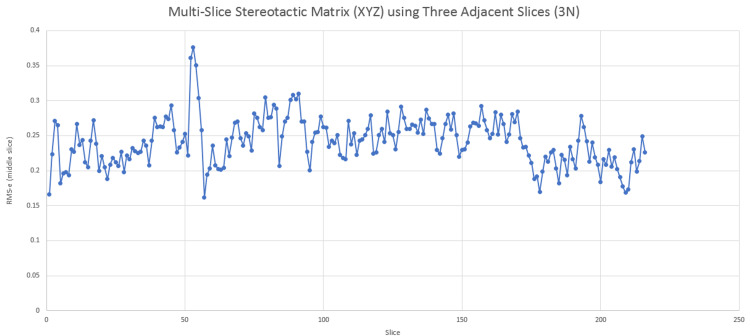
Utilization of the multi-slice stereotactic matrices (ms-SM) for three adjacent slices in a 3N localizer system Root Mean Square error (RMSe) is computed for all points in the middle slices by using the single-slice stereotactic matrix (ss-SM) and then comparing those results to the ms-SM. The results demonstrate a consistently low error (<0.4mm). Therefore, multi-slice computations can be adopted to subsets of slices, adjacent slices, or for the whole volume of slices.

Historically, single-slice stereotactic localization computes the ss-SM. This method is the cornerstone of frame-based stereotactic localization. Previously, we have shown that the use of overdetermined equations for ss-SM minimizes errors by using all the information in a single slice. While errors related to $x, y$ are minimized, errors related to $z$ are generally dependent on the minimum number of values needed to solve for the ss-SM. However, given the advance in technology, including the improvement in speed and imaging quality, volumes of data may contain a low degree of noise and movement. Making this assumption, multi-slice stereotactic localization may be performed. Examples of noise that may negatively affect multi-slice techniques include tremor during the scanning, image inhomogeneity, or the absence/mislabeling of sequential images. Careful analysis of the image set is very important before using multiple slices. Interestingly, multi-slice techniques can be flexibly computed and utilized for either adjacent slices or all slices in the volume. Figure 4 demonstrates a case where ms-SM used three adjacent slices resulting in low (<0.4mm) Root Mean Square error (RMSe) for the middle of the three slices throughout the volume. 

**Figure 5:**
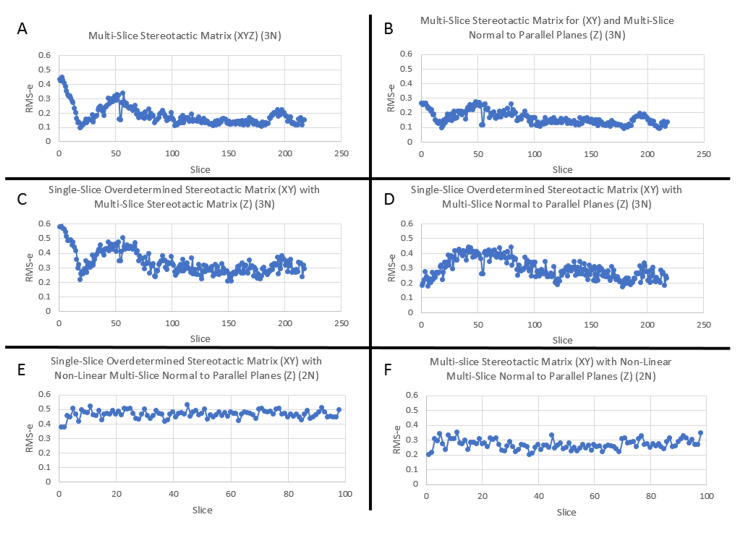
Example of single case studies using single-slice, multi-slice, or combination solutions The results of the tests were compared to ss-SM and Root Mean Square error (RMSe) was computed for each slice in the volume. For 2N systems, six points per slice were used to compute RMSe. For 3N systems, nine points per slice were used to compute RMSe. Multi-slice stereotactic matrix (ms-SM) can be used to compute XYZ data throughout the volume, here demonstrating an oscillation (A). Also, ms-SM can be used to compute XY and this can be combined with multi-slice normal to parallel planes (ms-nPP) for Z (B), which has slightly less error than A. Alternatively, single-slice stereotactic matrices (ss-SM) can be used on each slice for XY and combined with ms-SM (C) or ms-nPP (D) for Z. Also, ss-SM or ms-SM for XY can be used with non-linear ms-nPP for Z on parallel bipanel 2N systems (E-F).

With the addition of ms-SM and ms-nPP to ss-SM, multiple methods can be utilized for stereotactic localization. Some of these combinations were tested, and the results were compared to a three N-localizer system ss-SM for XYZ generating RMSe for each slice in the volume. First, ms-SM can be used to compute XYZ data throughout the volume, here demonstrating an initial sinusoidal oscillation (Figure 5A). The nature of the changing error is uncertain but has been observed in other case samples and may be related to an acceleration/deceleration or tremor during the imaging acquisition. Also, ms-SM can be used to compute XY, and this can be combined with ms-nPP for Z (Figure 5B), which has slightly less error than Figure 5A. Alternatively, overdetermined ss-SM can be used on each slice for XY and combined with ms-SM (Figure 5C) or ms-nPP (Figure 5D) for Z. Also, overdetermined ss-SM or ms-SM for XY can be used with non-linear ms-nPP for Z for a parallel bipanel 2N system (Figure 5E-5F). These data demonstrate that single-slice and multi-slice stereotactic localization are techniques that can be utilized in conjunction with each other. In particular, ss-SM for converting $u, v$ to $x, y$ are attractive as they incorporate patient movement on a slice-by-slice basis only with the disadvantage of needing to carry multiple matrices. Multi-slice techniques (ms-nPP or ms-SM) may be highly advantageous, especially to compute $z$.

**Figure 6:**
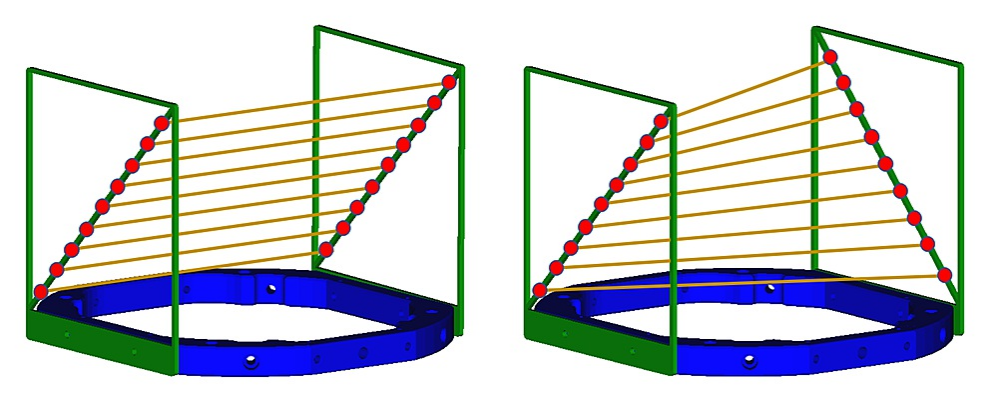
Representation of parallel (left) versus anti-parallel (right) bipanel N-localizer Points (red dots) along the diagonal bars can be followed in the vertical direction. The parallel bipanel contains diagonal bars that are parallel, and the points form lines that are also parallel and all coplanar. The anti-parallel bipanel contains points that form bars and lines that are not parallel and non-coplanar. Because of this relationship, the parallel bipanel requires a non-linear solution for multi-slice normal to parallel planes (ms-nPP), whereas the anti-parallel bipanel can be solved via linear solutions for ms-SM and ms-nPP.

Importantly, we have developed a new novel non-linear method that can be utilized to generate ms-nPP for a parallel bipanel N-localizer system. This non-linear method can also be used outside of the parallel bipanel. This solution is necessary for a parallel bipanel due to the coplanar points and thereby the difficulty using standard mathematical techniques, as previously noted. While one could alternatively use a geometric approach to compute vertical rod positions, we have found this approach to be more sensitive to errors. From a manufacturer standpoint, a simple solution is reversing one of the N-localizer panels to form an anti-parallel bipanel (Figure 6), as previously published [2]. Using an anti-parallel bipanel, one can observe that the N-bars have flipped orientation, making the diagonal bars skew. In addition, the lines generated for each slice are skew, not parallel, and non-coplanar.

**Figure 7:**
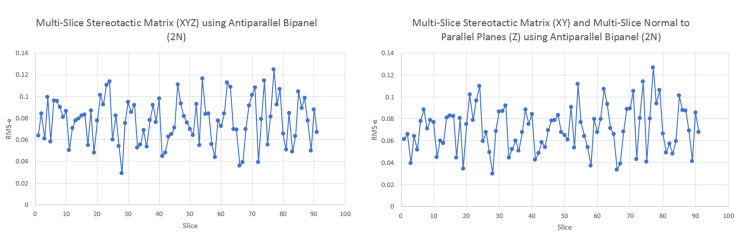
Anti-parallel bipanel phantom testing using multi-slice linear techniques Here, we utilized both multi-slice stereotactic matrix (ms-SM) and multi-slice normal to parallel planes (ms-nPP) and computed Root Mean Square error (RMSe) compared to single-slice stereotactic matrices (ss-SM) results.  We can observe a relatively low RMSe (<0.15mm) demonstrating good functionality of these techniques in the setting of the anti-parallel bipanel.

Mathematically, the anti-parallel bipanel affords the option for all of the multi-slice computations. As an example, we computed the ms-SM and ms-nPP for a phantom case localized using maximally stable extremal regions (MSER) for blob detection, which may introduce noise. One can see that the RMSe are less than 0.15mm, demonstrating that the multi-slice methods using linear computations are feasible for the anti-parallel bipanel (Figure 7). 

## Conclusions

Single-slice and multi-slice techniques are presented as important tools that can be used for stereotactic localization. Multi-slice solutions can be used for extrapolation to adjacent slices and, importantly, may improve solutions for computing the z-axis for 2N and 3N N-localizer systems. Also, the authors have developed a new novel non-linear method that can be used for parallel bipanel systems. Multi-slice stereotactic techniques assume the stability of adjacent slices related to the patient and imaging system during imaging acquisition. The optimal combination of methods will be the topic of future study and should be tested for a given configuration that may be dependent on the localizer system, the accuracy of the image acquisition, and the desired target. 
